# Diving deep into uterine layers: ADC and IVIM at 0.55-T MRI

**DOI:** 10.1186/s41747-026-00815-x

**Published:** 2026-09-17

**Authors:** Smiti Tripathy, Milauni Desai, Maximilian Lindholz, Lieselotte Kratzsch, Vignesh V. Pai, Franziska Mathis-Ullrich, Katharina Breininger, Jana Hutter

**Affiliations:** 1 Smart Imaging Lab, Radiologisches Institut, Universitätsklinikum Erlangen, Friedrich-Alexander-Universität Erlangen-Nürnberg (FAU), Erlangen, Germany; 2 Radiologisches Institut, Universitätsklinikum Erlangen, Friedrich-Alexander-Universität Erlangen-Nürnberg (FAU), Erlangen, Germany; 3 Department of Radiology, Charité Universitätsmedizin Berlin, Berlin, Germany; 4 Berlin Institute of Health, Berlin, Germany; 5 Department of Artificial Intelligence in Biomedical Engineering, Friedrich-Alexander-Universität Erlangen-Nürnberg (FAU), Erlangen, Germany; 6 Center for AI and Datascience, Julius-Maximilians-Universität Würzburg, Würzburg, Germany; 7 Institute for Information Processing, Leibniz University Hannover, Hannover, Germany

**Keywords:** Adenomyosis, Biomarkers, Diffusion magnetic resonance imaging, Endometrium, Myometrium

## Abstract

**Objective:**

To investigate and analyze the microstructure of uterine layers and to evaluate the feasibility and physiological variability of uterine diffusion metrics at 0.55-T MRI in healthy volunteers and participants with adenomyosis.

**Materials and methods:**

In this prospective study, 106 women, including healthy controls without (*n* = 47) and with hormonal contraception (*n* = 37), and patients diagnosed with adenomyosis (*n* = 22), underwent uterine diffusion MRI at 0.55 T. Apparent diffusion coefficient (ADC) and intravoxel incoherent motion (IVIM) were modeled using nine *b*-values in the myometrium, junctional zone, and endometrium. Quantitative intergroup differences, intragroup comparisons, and anterior–posterior wall analyses were performed. Evaluation of scan repetitions, intra-annotator and inter-annotator variability, and IVIM fit quality assessments were performed in subcohorts.

**Results:**

The intergroup analysis was performed on 106 women aged 27.2 ± 5.9 years (mean ± standard deviation) without bowel preparation or antispasmodics. A reduced median myometrium ADC (*p* = 0.024), reduced endometrial mean diffusivity (*p* = 0.017), and increased perfusion fraction (*p* = 0.014) were observed in the cohort with adenomyosis compared to healthy volunteers. Interindividual and intra-individual analysis of healthy volunteers demonstrated increased diffusivity in the endometrium during the luteal phase (*p* = 0.024). Additionally, differences in diffusion and IVIM parameters were observed in adenomyosis participants undergoing hormonal therapy. Repeat measurements, intra-annotator and inter-annotator agreement, and IVIM metric stability demonstrated good consistency.

**Conclusion:**

This study provides early evidence that uterine diffusion and IVIM at 0.55 T enable robust, non-invasive characterization of uterine microstructure sensitive to menstrual phase, hormonal status, and adenomyosis, providing insight into uterine physiology and pathology.

**Key Points:**

***Question*** Can 0.55-T MRI characterize functional changes associated with adenomyosis compared with healthy uteri?

***Findings*** 0.55-T uterine diffusion MRI without bowel preparation quantified uterine microstructure, with layer-specific ADC and IVIM distinguishing adenomyosis, hormonal effects, menstrual phase, and anterior–posterior differences.

***Relevance statement*** Quantitative diffusion and IVIM metrics at 0.55-T distinguish adenomyosis from healthy tissue through reduced myometrial ADC, endometrial diffusivity, and increased perfusion fraction. These biomarkers hold promise in clarifying diagnosis and supporting future studies assessing hormonal therapy response and treatment monitoring.

**Graphical Abstract:**

**Figure:**
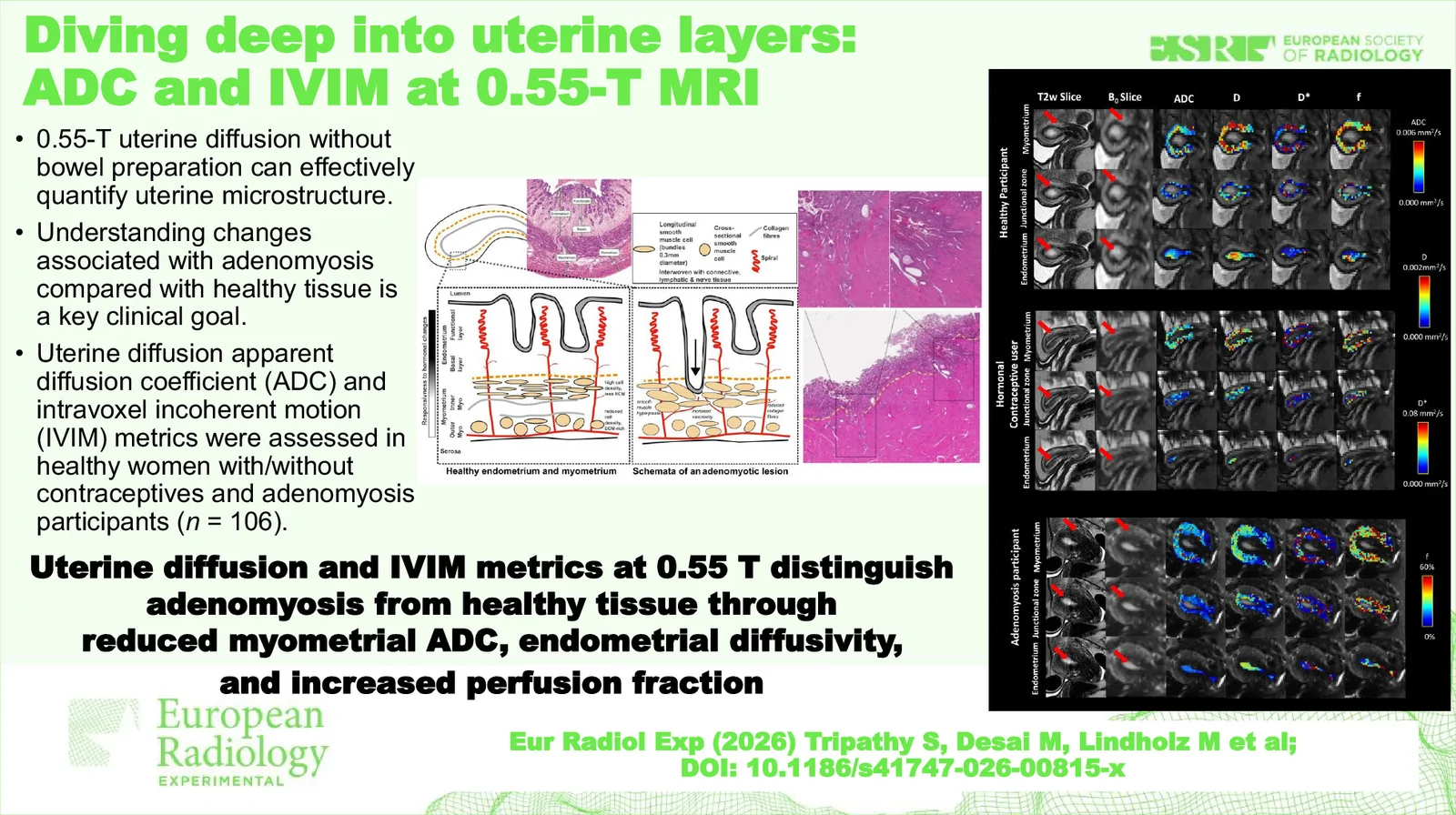

## Background

**Fig. 1:**
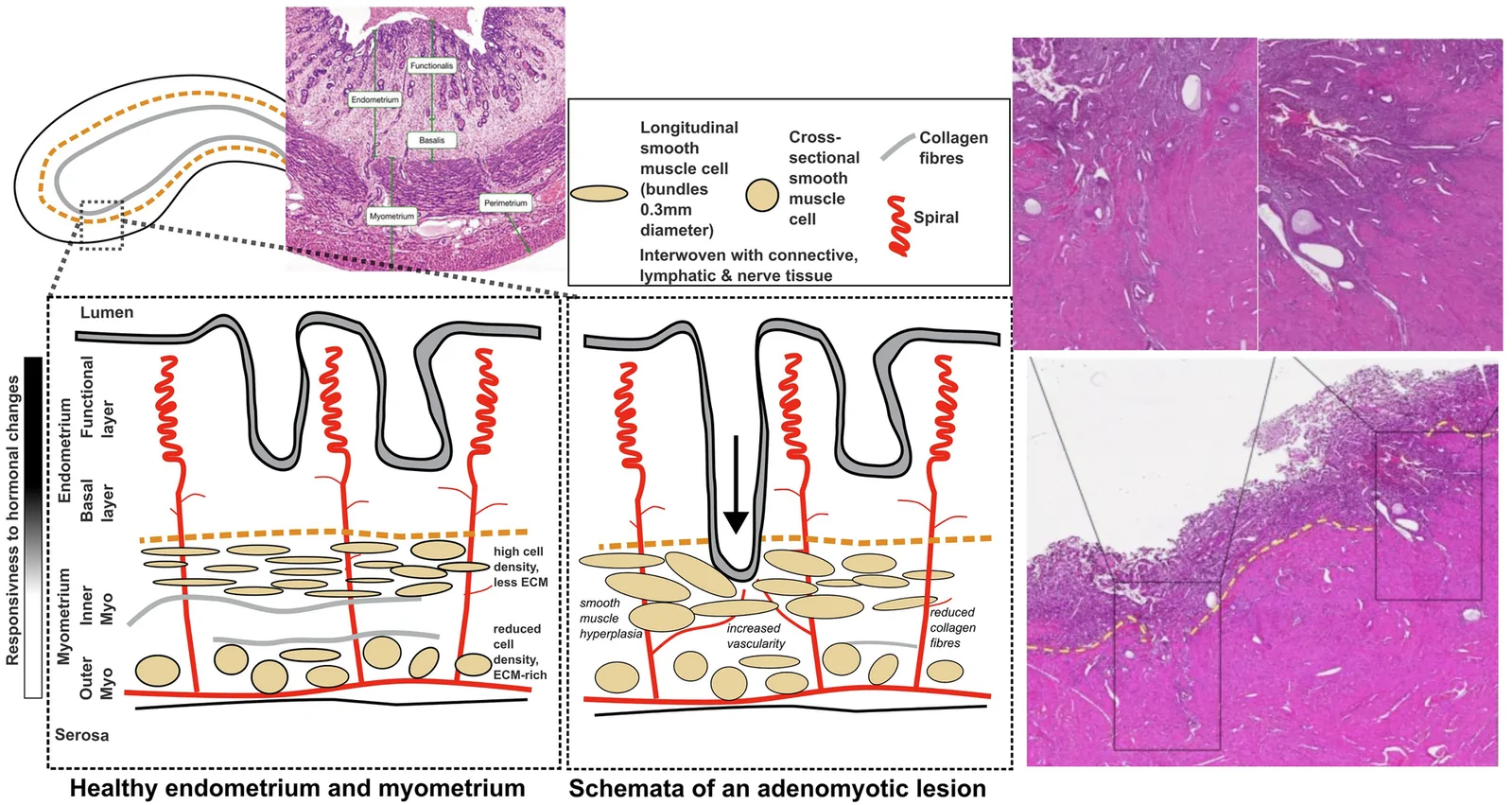
Schemata of microstructural changes associated with adenomyosis in the uterine layers in comparison with healthy myometrium and endometrium

Among magnetic resonance imaging (MRI) techniques, diffusion-weighted imaging (DWI) offers tissue microstructural insights that complement morphological assessment. This is of interest both for research and clinical settings, for example, in complex multi-layered organs such as the uterus, where the delicate microstructure of the layers is tightly linked to both healthy uterine physiology [1] and gynecological pathology, including malignant disease [2] and benign disorders such as adenomyosis, endometriosis, and fibroids. The uterus is a fibromuscular organ consisting of three layers: myometrium, junctional zone, and endometrium. The myometrium consists of smooth muscle bundles [3], the junctional zone is composed of densely packed muscle fibers with relatively low water content [4], and the endometrium is characterized by a mucous membrane. This study focuses on adenomyosis as a benign uterine condition with a complex pathway of events that affect the microstructure of the uterine layers. Adenomyosis is associated with dysmenorrhea, menorrhagia, and sub-fertility, characterized by ectopic invasion of endometrial glands and stroma into the myometrium [5]. These changes are accompanied by increased vascularity, altered collagen composition, and increased stiffness, as schematically illustrated in Fig. 1.

Parametrized models such as the mono-exponential apparent diffusion coefficient (ADC) and intravoxel incoherent motion (IVIM) can capture alterations in hindered diffusion and microvascular pseudoperfusion expected in normal uterine layers and gynecologic diseases. While clinically most often ADC analysis is conducted, due to the reduced requirements in terms of acquisition parameters and thus time, increasingly complex models in research settings allow even deeper insights. IVIM [6] has shown the ability to disentangle tissue diffusivity and microcirculation-related perfusion, while diffusion kurtosis imaging has revealed menstrual cycle–dependent microstructural changes in the uterine layers [7]. Diffusion tensor imaging with HARDI-type protocols has further demonstrated directional microstructural organization within the myometrium and junctional zone [8], although such approaches remain technically challenging in the pelvis.

To date, most uterine diffusion MRI studies have focused on healthy volunteers not using hormonal contraceptives, or on small cohorts diagnosed with pathologies. Across these studies, variations in ADC, fractional anisotropy, kurtosis metrics, and IVIM parameters have been reported with respect to menstrual cycle phase, age, and disease status, reflecting the dynamic changes within the uterus [7]. IVIM biomarkers have also demonstrated utility in clinical contexts, including treatment planning and monitoring for MRI-guided high-intensity focused ultrasound fibroid ablation [9] and improved characterization of endometrial cancer [10]. Combined IVIM and diffusion kurtosis imaging modeling has shown additive sensitivity to subtle endometrial and junctional zone changes [2].

While abdominal MRI has typically been conducted at 1.5 or 3 T, recent re-emergence of the lower field strength of 0.55 T has particularly positively impacted body MRI applications [11]; reduced susceptibility and specific absorption rate as well as longer T2* [12]. These are especially attractive for clinical pelvic imaging, where higher magnetic field strengths are prone to geometric distortion arising from air-tissue interfaces, typically requiring preparation with antispasmodic agents. Reducing the need for such preparation can reduce examination time, burden, and overall preparation for clinical pelvic MRI. While DWI benefits from reduced susceptibility and longer T2*, which can lessen echo-planar imaging (EPI) distortion and T2* shine-through, the lower signal-to-noise ratio (SNR) remains a major limitation. This is particularly difficult for DWI acquisitions operating close to the noise floor, and for models relying on high *b*-values, multi-echo readouts, or high spatial resolution. In such analysis, reduced SNR magnifies bias in ADC estimation and can amplify IVIM instability, especially for pseudodiffusivity.

Uterine MRI especially remains challenging due to various sources of motion, particularly uterine peristalsis and bowel peristalsis at all field strengths. Published uterine data at 0.55 T are scarce; most low-field diffusion reports to date focus on the abdomen or lung. Therefore, there is a gap in the evidence regarding the feasibility and precision of uterine ADC/IVIM at low-field, and the sensitivity of these models to adenomyosis under low-SNR conditions. Establishing how ADC and IVIM behave in both healthy uterine layers and, in particular, in adenomyosis is a prerequisite to using microstructural MRI more widely for quantitative gynecologic imaging. Furthermore, several pathophysiological features have been shown to vary with location [13], and, for example, adenomyosis involving the posterior uterine wall was shown to be more frequent in severe stages of endometriosis [14], whereas anterior wall involvement has been linked to dysuria and bladder in endometriosis, calling for a location-specific analysis.

We address these gaps by acquiring standardized low-field uterine DWI in both healthy volunteers and patients with adenomyosis, estimating ADC and IVIM parameters with noise-aware fitting. We test: (1) whether layer-resolved diffusion metrics can be reproducibly quantified at 0.55 T, (2) whether adenomyosis exhibits a detectable diffusion and IVIM signature relative to healthy uteri despite low-field SNR constraints, (3) whether menstrual phase and hormonal contraceptive status produce quantifiable changes in diffusion metrics, (4) whether anterior–posterior uterine wall differences are reflected in diffusion parameters, and (5) whether diffusion metrics remain consistent across repeated scan time points (test–retest repeatability). We thereby especially evaluate the feasibility and physiological variability of uterine diffusion metrics at low-field MRI.

## Materials and methods

### Study population and MRI protocol

**Fig. 2:**
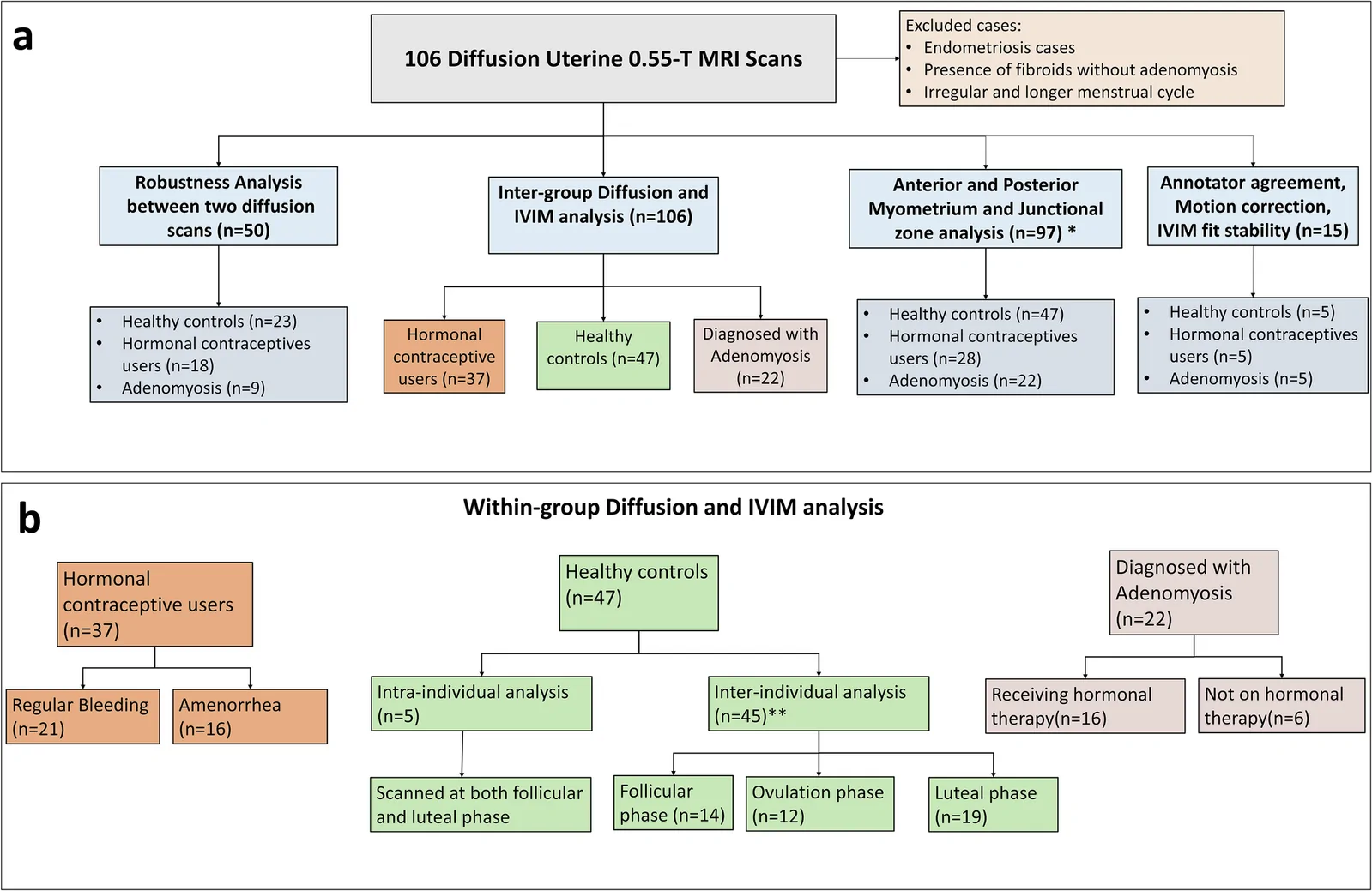
**a** Overview of the study participants included for diffusion and intravoxel incoherent motion (IVIM) analysis, test–retest repeatability assessment, and anterior–posterior myometrium and junctional zone analysis. * *n* = 9 lacking a visible or few voxels of endometrium excluded as a reliable split of the layers could not be performed. **b** Within-group analysis of the three cohorts divided on the basis of menstrual cycle state and hormonal therapy treatment. ** *n* = 2 participants on menstruation excluded for interindividual analysis of healthy controls

In this prospective study, uterine MRI was performed between October 2024 and October 2025 on a clinical 0.55-T Magnetom Free.Max scanner (Siemens Healthineers, Forchheim) using a 9-channel contour-M coil and an embedded 6-channel spine coil in supine position in 106 women after written informed consent (the medical faculty’s ethics committee approved the study: 23-444-BM). No bowel or bladder preparation was performed, and no antiperistalsis drugs or contrast agents were administered. The participants were divided into three study groups: healthy volunteers with regular menstruation (25–40 days), healthy volunteers using hormonal contraceptives or intrauterine devices, and women clinically diagnosed with adenomyosis (*via* laparoscopy or histopathology or ultrasound). Participants presenting with both adenomyosis and coexisting uterine fibroids were included in the adenomyosis cohort. Each group was further subdivided for within-group analysis. The healthy control group was categorized according to the self-reported phase of the menstrual cycle during the scan into follicular, ovulation, and luteal. In addition, five healthy volunteers were scanned longitudinally during both the follicular and luteal phases. The hormonal contraceptive group was subdivided into participants with regular bleeding or withdrawal bleeding and those with amenorrhea. Participants diagnosed with adenomyosis were divided based on whether they were receiving hormonal therapy at the time of scan. Additionally, the adenomyosis cohort was subdivided by an experienced radiologist (3 years of experience in urogenital and gynecological imaging) into the following MRI-based classifications: type I (intrinsic focal adenomyosis), type II (extrinsic focal adenomyosis), type III (intramural focal adenomyosis), IVa (diffuse adenomyosis with unilateral myometrium involvement), and type IVb (diffuse adenomyosis with bilateral myometrium involvement) on sagittal T2-weighted turbo-spin echo (TSE) images. The exclusion criteria included a diagnosis of endometriosis, presence of fibroids without adenomyosis, irregular menstrual cycles over the preceding months, and current pregnancy. Figure 2 presents the detailed overview of the study participants for the quantitative diffusion analysis.

**Fig. 3:**
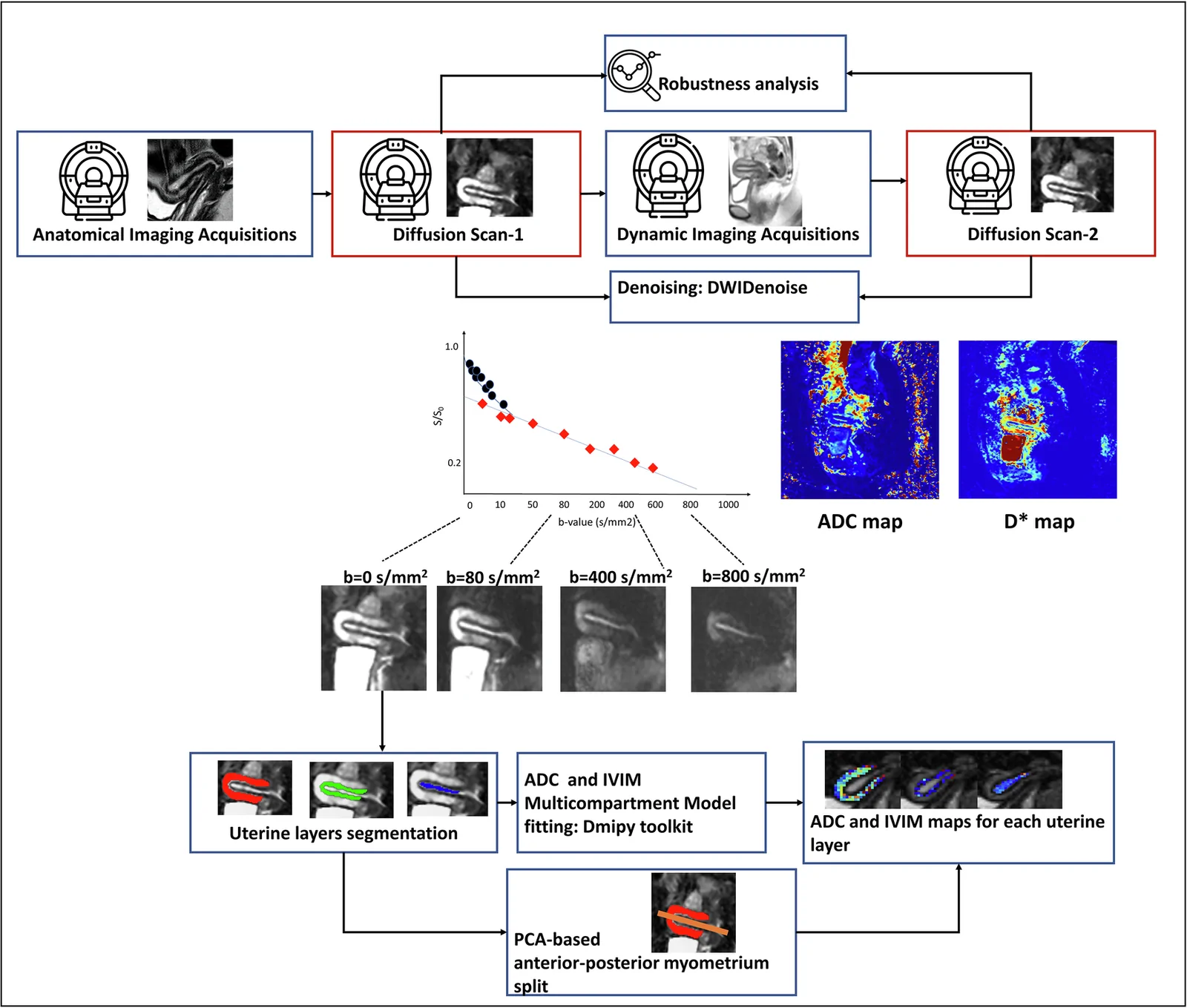
Processing pipeline from acquisition to analysis. From top to bottom: acquisition schemata including repetition scans and applied pre-processing. Middle: illustrative data at the various *b*-values on a mid-sagittal slice of an exemplary case, together with mean values against *b*-value and whole slice apparent diffusion coefficient (ADC) and pseudodiffusivity (D*) maps. Bottom: Performed analysis from uterine layer segmentation to model fitting and split into the anterior and posterior aspects of the uterus

Sagittal 2D single-shot echo-planar diffusion-weighted imaging (EPI-DWI) was acquired following anatomical T2-weighted imaging, and was repeated after dynamic cine MRI sequences (Fig. 3). The time interval between the first and second diffusion acquisitions was approximately 20 min. Imaging parameters were as follows: field of view = 302 × 298 mm²; voxel size = 2.7 × 2.7 × 5 mm³; number of slices = 25; repetition time = 5,300 ms, echo time = 155 ms; and bandwidth = 1,204 Hz/pixel. The longer echo time resulted mainly from the reduced gradient strength available at the employed commercial 0.55-T system in combination with the preferred higher in-plane resolution. Diffusion weighting was applied using nine *b*-values (0, 10, 50, 80, 200, 400, 600, 800, and 1,000 s/mm²) applied to all three orthogonal directions, performed and stored individually, to allow robust IVIM mapping. No fat suppression was applied. No *b*-value averaging was performed to ensure clinical feasibility in terms of acquisition time while still enabling the larger number of *b*-values required for advanced diffusion analysis. No manual shimming was performed. The acquisition time for each DWI was 2:38 min.

### Data processing and quantitative analysis

The acquired datasets were first denoised using random matrix theory [15]. The myometrium, junctional zone, and endometrium were manually segmented across all uterine slices using the *b* = 0 s/mm² volume as anatomical reference, performed by one annotator and subsequently reviewed and refined by a second annotator. Diffusion modeling was performed on non-motion-corrected data by applying a mono-exponential model to estimate voxel-wise ADC and a multi-compartment Gaussian diffusion model to estimate IVIM parameters such as diffusivity (D (mm^2^/s)), pseudodiffusivity (D* (mm^2^/s)), and perfusion fraction (f (%)) using the Dmipy package [16]. Thereby, implicitly averaging of direction-dependent signals within the same *b*-value is performed. Default Dmipy constraint parameters were employed, and full fitting was performed. Additionally, principal component analysis–based midline extraction was performed on the endometrium along its long axis to obtain anterior–posterior wall-specific values in the myometrium and junctional zone.

A subset of 15 randomly selected cases (healthy controls, *n* = 5; hormonal contraceptive users, *n* = 5; adenomyosis, *n* = 5) was resegmented by the first annotator for intra-annotator analysis and, in addition, by an additional annotator for inter-annotator variability. This subset was, in addition, motion-corrected with the ANTs toolkit [17]. Motion correction was performed by registering diffusion-weighted images at higher *b*-values to the *b* = 0 s/mm² reference volume using symmetric normalization deformable registration. IVIM fit curve stability was assessed by comparing the fitted IVIM model to the measured mean diffusion signal at each *b*-value.

### Statistical analysis

Statistical analysis was performed using Python packages pingouin (v0.5.5) and scipy.stats. Mean and median values of ADC and each IVIM parameter (D, D*, f) were extracted from the voxel-wise measurements to assess intergroup differences. Normality of both mean and median data distribution was assessed separately for each comparison using the Shapiro–Wilk test. Data with a normal distribution are expressed as mean ± standard deviation, whereas non-normally distributed data are reported as median (minimum–maximum). Depending on data normality and homogeneity of variances, either Welch’s *t*-test or the Mann–Whitney *U* test was used to analyze intergroup and within-group differences. False discovery rate correction (Benjamini–Hochberg) was applied to intergroup comparisons. Anterior–posterior wall analyses and within-group comparisons were reported as exploratory. Bland–Altman analysis was performed to assess the robustness and repeatability of the two acquired diffusion scans. Intra-annotator and inter-annotator agreement for segmentation was assessed using two-way intraclass correlation coefficients (ICCs). IVIM model fitting and stability before and after motion correction registration were assessed using R^2^ and the percentage of variability for each parameter. *p* < 0.05 was considered statistically significant.

## Results

### Intergroup quantitative ADC and IVIM analysis

**Fig. 4:**
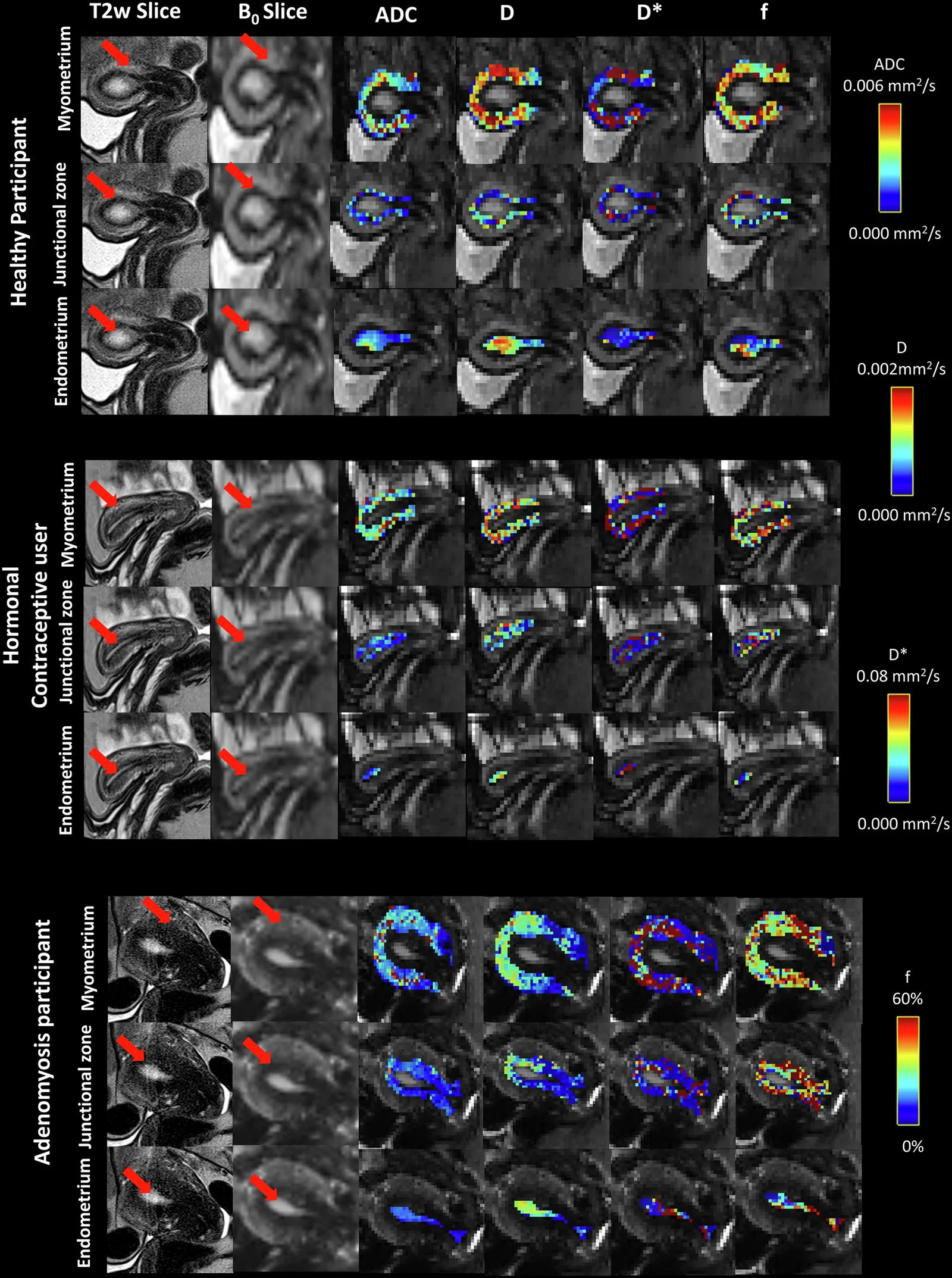
T2-weighted uterine mid-sagittal slice, apparent diffusion coefficient (ADC), diffusivity (D), pseudodiffusivity (D*) and perfusion fraction (f) maps of the three uterine layers, overlaid on the corresponding *b* = 0 s/mm² images, are shown for a healthy volunteer without and with contraceptive usage and a participant with adenomyosis. Shown in red arrows are representative uterine layers

Diffusion data were successfully acquired and analyzed in all 106 participants (age: 27.2 ± 5.9 years, mean ± standard deviation; range: 18–49 years). Table 1 describes the study participants’ characteristics. Figure 4 shows ADC and IVIM maps for exemplary cases of all three cohorts: a healthy volunteer, a hormonal contraceptive user, and a participant with adenomyosis depicting the layer-specific changes. Figure S1 illustrates the averaged quantitative results and the density distribution across the three cohorts and uterine layers. Table 2 summarizes the mean and median values of ADC and IVIM metrics for each uterine layer and each cohort.

**Table 1:** Study participants characteristics

Characteristics	Value
Number of participants	106
Age, years	27.2 ± 5.9 [18–49]
Weight, kg	65.2 ± 12.7 [43–115]
Height, cm	166.3 ± 7.2 [149–185]
Adenomyosis* (diagnosis modality)	Ultrasound: 7 Laparoscopy: 7 Histopathologic confirmation: 2 MRI: 6
MRI classification of adenomyosis	Type I (intrinsic focal adenomyosis): 13 Type II (extrinsic focal adenomyosis): 2 Type III (intramural focal adenomyosis): 1 Type IVa (diffuse adenomyosis): 1 Type IVb (diffuse bilateral myometrium adenomyosis): 1 Other: 4**

Numerical values are reported as mean ± standard deviation [range]

* *n* = 5 adenomyosis cases with coexisting fibroids

** Four cases showed imaging features of adenomyosis but did not meet criteria for classification into the predefined subtypes and were therefore categorized as other

**Table 2:** Comparison of the mean and median apparent diffusion coefficient (ADC), diffusivity (D), and pseudodiffusivity (D*) and perfusion fraction (f) across healthy controls, hormonal contraceptive users, and participants with adenomyosis in the myometrium, junctional zone, and endometrium

Value	ADC (× 10^-^^3^ mm^2^/s)		D (× 10^-^^3^ mm^2^/s)		D* (× 10^-^^3^ mm^2^/s)		f (%)	
	Mean	Median^†^	Mean	Median^†^	Mean	Median^†^	Mean	Median^†^
Myometrium								
Healthy control	5.9 ± 2.0	2.8 ± 0.8	1.1 ± 0.2	1.1 ± 0.3	48.8 ± 9.4	40.9 ± 24.5	42.0 ± 5.0	42.1 ± 5.0
Hormonal contraceptive users	4.7 [27.3–2.7]	2.7 ± 1.5	1.1 ± 0.3	1.1 ± 0.3	47.9 ± 10.5	39.8 ± 23.0	40.2 ± 5.4	40.1 ± 6.9
Adenomyosis	5.1 ± 1.9	2.3 ± 0.5*	0.9 ± 0.2	0.9 ± 0.3	49.2 ± 9.2	42.1 ± 24.5	41.0 ± 4.7	40.4 ± 5.6
Junctional zone								
Healthy control	1.7 ± 0.5	1.4 ± 0.3	0.7 ± 0.2	0.8 ± 0.2	33.7 ± 12.3	16.0 ± 20.4	25.7 [36.5–4.3]	24.4 ± 7.8
Hormonal contraceptive users	1.6 ± 0.4	1.3 ± 0.3	0.8 ± 0.2	0.8 ± 0.2	31.5 ± 11.5	13.8 ± 14.8	24.7 ± 6.6	21.8 ± 9.3
Adenomyosis	1.5 ± 0.3	1.4 ± 0.3	0.7 ± 0.2	0.7 ± 0.2	35.1 ± 11.6	19.3 ± 15.5	28.7 ± 6.1	27.3 ± 9.8
Endometrium								
Healthy control	1.7 [7.9–0.8]	1.5 ± 0.3	1.0 ± 0.2	1.0 ± 0.2	33.1 ± 13.2	17.0 ± 22.3	22.8 [45–12.9]	20.0 ± 8.4
Hormonal contraceptive users	1.6 [7.2–0.6]	1.6 ± 0.4	0.9 ± 0.2	0.9 ± 0.2	37.4 ± 16.1	23.3 ± 21.6	28.7 ± 7.7**	26.4 ± 10.2*
Adenomyosis	1.6 [5.7–0.9]	1.4 ± 0.3	0.8 ± 0.2*	0.8 ± 0.2*	36.1 ± 12.6	21.0 ± 20.3	27.9 ± 8.0*	25.9 ± 9.3*

Numerical values are reported as mean ± SD (standard deviation) or median [maximum–minimum range]

* *p* < 0.05, ** *p* < 0.01, compared with healthy controls

† For each ADC and IVIM parameter, per-participant median values were computed, and overall group statistics are reported as mean ± SD

Mean ADC was higher in the myometrium compared to the endometrium and junctional zone (*p* < 0.001) across all groups. The adenomyosis group exhibited a lower mean of per-participant median ADC (2.3 ± 0.5 × 10^-^^3^ mm^2^/s) in the myometrium compared to healthy controls (2.8 ± 0.8 × 10^-^^3^ mm^2^/s, *p* = 0.024).

In the endometrium, adenomyosis was correlated to a significant reduction in both mean (0.8 ± 0.2 × 10^-^^3^ mm^2^/s) and mean of per-participant median D values relative to healthy uteri (1.0 ± 0.2 × 10^-^^3^ mm^2^/s, *p* = 0.017), and an increased perfusion fraction f (mean of per-participant median: *p* = 0.027; mean: *p* = 0.044). Furthermore, endometrial perfusion was significantly higher in hormonal contraceptive users (mean of per-participant median: 26.4 ± 10.2%; mean: 28.7 ± 7.7%) compared to healthy controls (mean of per-participant median: 20.4 ± 8.4%, *p* = 0.014; mean: 22.8 (45–12.9) %, *p* = 0.002).

### Anterior and posterior uterine wall analysis

Cases (*n* = 9) with less than five endometrium voxels were excluded for the analysis of the anterior–posterior uterine wall, as a reliable principal component analysis–based split could not be performed. Anterior and posterior walls of myometrium and junctional zone differences in ADC and IVIM metrics are depicted in Fig. S2. As demonstrated, the mean ADC in the posterior wall of the myometrium of hormonal contraceptive users increased by 12.6% (mean difference: +0.8 × 10^-3^ mm^2^/s) compared to the anterior wall, whereas the cohort with adenomyosis showed an 11% (mean difference: -0.6 × 10^-3^ mm^2^/s) decrease in the posterior relative to the anterior wall. Hormonal contraceptive users exhibited a lower mean ADC in the anterior wall (*p* = 0.041) compared to healthy controls, with no significant difference in the posterior wall. Diffusivity D in the myometrium of participants with adenomyosis was 6.4% higher in the anterior wall, with reduced values observed in both walls compared to healthy controls (anterior: *p* = 0.035; posterior: *p* = 0.024). Similarly, in the junctional zone, mean ADC in the anterior wall was 6.3% higher than in the posterior wall in participants with adenomyosis. Perfusion fraction was reduced in hormonal contraceptive users compared to the adenomyosis cohort (*p* = 0.032) in both the walls.

### Interindividual differences and analysis

#### Healthy controls

**Fig. 5:**
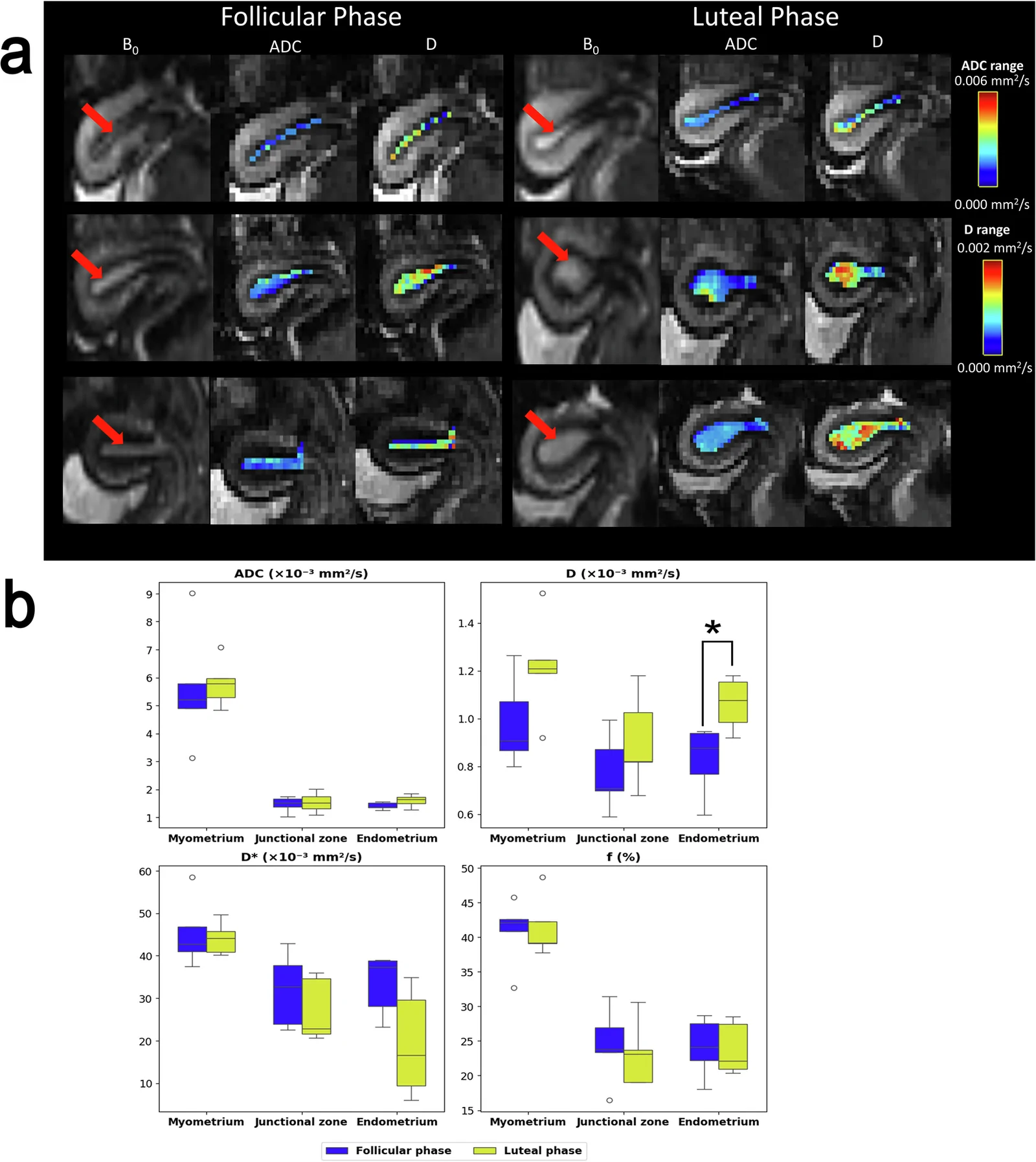
**a** Apparent diffusion coefficient (ADC) and diffusivity (D) maps of the endometrium in three healthy participants scanned during the follicular and luteal phases. Maps are overlaid on the corresponding *b* = 0 s/mm² images. **b** Boxplots of ADC and intravoxel incoherent motion (IVIM) parameters (diffusivity (D) and pseudodiffusivity (D*) and perfusion fraction (f)) across the three uterine layers for five participants scanned during the follicular and luteal phases. The red arrow denotes the endometrial layer. * *p* < 0.05

Figure 5a presents representative ADC and D maps of the endometrium from three healthy volunteers scanned during the follicular and luteal phases. Intra-individual boxplots for five participants scanned during the follicular and luteal phases are shown in Fig. 5b. Table 3 shows the mean quantitative interindividual diffusion metrics at follicular, ovulation, and luteal phases. Endometrial thickening (Fig. 5a) was observed for all five volunteers from the follicular phase to the luteal phase, with increased mean ADC and D (*p* = 0.024) in the luteal phase relative to the follicular phase. A similar trend was observed in the interindividual analysis, with an increase in endometrial D during the luteal phase (*p* = 0.025).

**Table 3:** Apparent diffusion coefficient (ADC), diffusivity (D), and pseudodiffusivity (D*) and perfusion fraction (f) parameters for interindividual healthy participants scanned at the follicular, ovulation, and luteal phases

Value	ADC (× 10^-3^ mm^2^/s)	D (× 10^-3^ mm^2^/s)	D* (× 10^-3^ mm^2^/s)	f (%)
Myometrium				
Follicular phase	6.3 ± 3.0	1.1 ± 0.3	52.7 ± 11.0**	41.3 ± 4.9
Ovulation phase	5.6 ± 1.7	1.1 ± 0.2	48.3 ± 7.0	42.1 ± 5.3
Luteal phase	5.7 ± 2.0	1.1 ± 0.2	43.3 ± 7.0	42.7 ± 4.8
Junctional zone				
Follicular phase	1.7 ± 0.6	0.8 ± 0.2	34.5 ± 15.3	26.3 [34.7–4.3]
Ovulation phase	1.6 ± 0.3	0.7 ± 0.2	31.6 ± 10.1	26.2 ± 4.0
Luteal phase	1.7 ± 0.2	0.8 ± 0.2	30.5 ± 9.1	25.7 [41.3–19.0]
Endometrium				
Follicular phase	1.5 [7.9–0.5]	0.9 ± 0.3*	33.7 ± 15.3	24 ± 6.8
Ovulation phase	1.7 [5.0–1.1]	1.0 ± 0.1	34.0 ± 7.8	23.8 ± 5.0
Luteal phase	1.7 [3.7–1.3]	1.1 ± 0.2	28.9 ± 12.5	24.2 ± 7.0

Numerical values are reported as mean ± standard deviation or median [maximum–minimum range]

* *p* < 0.05

** *p* < 0.01

#### Adenomyosis

**Fig. 6:**
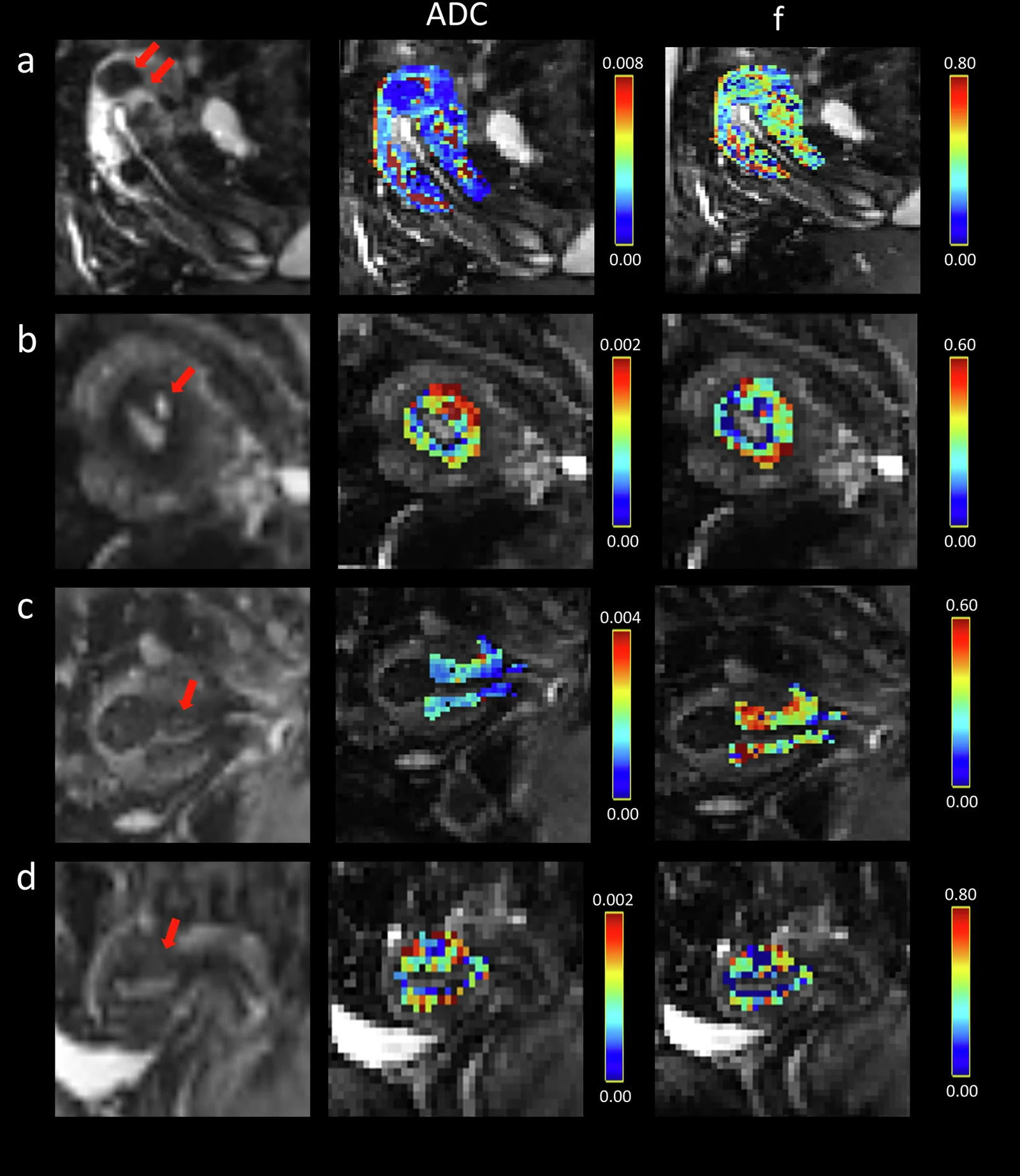
Apparent diffusion coefficient (ADC) and perfusion fraction (f) maps in the myometrium and junctional zone for four participants from the adenomyosis cohort. **a** Type I intrinsic and Type II extrinsic focal adenomyosis with an enlarged uterus and multiple fibroids (red arrow). **b** Type I focal adenomyosis, with a cyst in the junctional zone (red arrow). **c** Type III intramural focal adenomyosis, with multiple cysts in the junctional zone (red arrow). **d** Type I (focal) adenomyosis with focal thickening of the junctional zone in the posterior uterine wall (red arrow)

Figure 6 illustrates the ADC and perfusion maps of four cases from the adenomyosis cohort. Figure S3 shows the range of diffusion values for the subtyped adenomyosis cases. The adenomyosis group was further subdivided based on hormonal therapy, as presented in Table 4 with participants not receiving hormonal therapy exhibiting higher myometrial D (*p* = 0.015), increased junctional zone diffusion (*p* = 0.031), and higher endometrial ADC (*p* = 0.016) and D (*p* = 0.021) compared to participants treated with hormonal therapy.

**Table 4:** Apparent diffusion coefficient (ADC), diffusivity (D), and pseudodiffusivity (D*) and perfusion fraction (f) values participants with adenomyosis, grouped according to whether they were taking hormonal medication during the scan

Value	ADC (× 10^-3^ mm^2^/s)	D (× 10^-3^ mm^2^/s)	D* (× 10^-3^ mm^2^/s)	f (%)
Myometrium				
Hormone therapy	5.0 ± 2.0	0.9 ± 0.2	48.6 ± 9.8	41.7 ± 4.9
Not on hormone therapy	5.5 ± 1.7	1.1 ± 0.2*	50.9 ± 7.1	39.2 ± 3.7
Junctional zone				
Hormone therapy	1.5 ± 0.3	0.6 ± 0.2	35.4 ± 12.2	29.2 ± 6.7
Not on hormone therapy	1.8 ± 0.2*	0.8 ± 0.2	34.3 ± 9.8	27.3 ± 3.8
Endometrium				
Hormone therapy	1.4 ± 0.3	0.7 ± 0.2	38.2 ± 13.7	28.1 ± 8.2
Not on hormone therapy	2.7 ± 1.5*	1.0 ± 0.2*	30.7 ± 6.1	27.6 ± 7.1

Numerical values are reported as mean ± standard deviation

* *p* < 0.05

#### Hormonal contraceptive users

Table S1 shows the differences in diffusion values between participants undergoing regular menstruation and amenorrheic volunteers among the hormonal contraceptive users. Significant differences were detected only in endometrial D*, with higher values observed in amenorrheic participants (*p* = 0.008).

### Robustness analysis

The Bland–Altman plots in Fig. S4 for each diffusion and IVIM parameter across the three cohorts showed overall low mean differences (ADC myometrium - mean difference: -0.20 × 10^-^^3^ mm^2^/s, 95% limits of agreement: -6.31 to 5.92 × 10^-^^3^ mm^2^/s; D myometrium - mean difference: 0.02 × 10^-^^3^ mm^2^/s, 95% limits of agreement: -0.34 to 0.38 × 10^-^^3^ mm^2^/s). For D* in healthy participants in the junctional zone a mean difference of -4.27 × 10^-^^3^ mm^2^/s (95% limits of agreement: -23.92 to 15.38 × 10^-^^3^ mm^2^/s) was obtained, for hormonal contraceptive users in the myometrium a mean difference of 3.78 × 10^-^^3^ mm^2^/s (95% limits: -16.18 to 23.74 × 10^-^^3^ mm^2^/s) and for the adenomyosis cohort in the myometrium a mean difference of -5.57 × 10^-^^3^ mm^2^/s (95% limits: -46.39 to 35.25 × 10^-^^3^ mm^2^/s) was obtained.

Table S2 details the inter-annotator (ICC: 0.41–0.98) and intra-annotator (ICC: 0.57–0.99) agreement on the subset of *n* = 15 cases. The inter-annotator ICC for the endometrial F parameter in healthy subjects was 0.41, and the intra-annotator ICC for the endometrial D* parameter in adenomyosis subject was 0.57. Figure S5 presents the IVIM fit curve with that of the measured signal for three cases. The performed IVIM model demonstrated good stability with a mean ± standard deviation R^2^ value of 0.96 ± 0.02 (myometrium), 0.93 ± 0.04 (junctional zone), and 0.94 ± 0.02 (endometrium). Following motion correction, mean R² values were 0.95 ± 0.02 for myometrium, 0.86 ± 0.09 for junctional zone, and 0.96 ± 0.02 for the endometrium. Table S3 provides the percentage of variability before and after motion correction registration for each metric across the cohorts.

## Discussion

This study demonstrates the feasibility of performing quantitative layer-specific diffusion and IVIM analysis in the uterus *in vivo* at 0.55 T. The reduced susceptibility artifacts enabled clear visualization and quantification of uterine zonal characteristics without requiring bowel preparation, reducing acquisition burden in clinical practice. Adenomyosis exhibited distinct diffusion signatures: reduced myometrial ADC, reduced endometrial diffusivity, and increased perfusion fraction compared to healthy controls. Scan repetitions, intra-annotator and inter-annotator analysis, as well as fit quality assessments, demonstrated robust measurement reproducibility.

Mean and median diffusion metrics for intergroup comparisons were chosen, as they have been shown previously to be robust for uterine diffusion metrics [18]. The observed higher ADC values in this study are likely due to the inclusion of low *b*-values, which reflect combined diffusion and perfusion effects. Significant differences in ADC among uterine layers are consistent with previous reports [19]. Distinct diffusion and perfusion characteristics were observed across layers and cohorts, highlighting sensitivity to both hormonal and pathological alterations. A significant reduction in median ADC and a trend toward reduced mean ADC were observed in the myometrium of participants with adenomyosis compared to healthy myometrial tissue, supporting previously reported patterns in this cohort [20, 21]. Additionally, reduced myometrial D was observed in adenomyosis, potentially reflecting tissue micro-trauma and invasion of ectopic endometrial glands into the smooth myometrial layer [21–23]. No significant differences were identified in the ADC of the junctional zone between the adenomyosis and healthy controls. The trend of reduced ADC compared to prior reports [21] may be due to the inclusion of participants both on and off hormonal therapy. In line with this, a trend toward increased junctional zone ADC was observed in participants with adenomyosis not on hormonal therapy. A similar pattern was observed in the endometrium.

Significantly reduced D and increased perfusion compared to healthy endometrium were found, and this is likely due to increased angiogenesis and intensified abnormal vascularization in adenomyosis [24]. In addition, hormonal contraceptive users also showed significantly increased endometrial perfusion relative to healthy controls. This finding may be related to the duration of hormonal contraceptive usage, as early stages of usage are associated with stromal edema in the endometrium and thinning of blood vessels [25], with prolonged use leading to endometrial atrophy as observed on T2-weighted MRI [26]. Furthermore, depending on the type of hormonal contraceptive or intrauterine devices, previous studies [27] reported increased subendometrial vascularization and alterations in the endometrial vasculature. Given the mixed cohort in our study, comprising participants using different contraceptives and intrauterine devices, it is difficult to attribute the observed increase in endometrial diffusivity in amenorrheic participants compared to participants with regular menstrual cycles.

The observed differences in diffusion and IVIM parameters between the anterior and posterior walls of the myometrium and junctional zone in both participants with adenomyosis and hormonal contraceptive users might be linked to adenomyosis presenting a heterogeneous condition [28]. This spatial heterogeneity suggests regionally localized microstructural variation contributing to the anterior–posterior differences observed in diffusion and perfusion metrics.

The pattern of increased mean ADC across all three uterine layers from the follicular phase to the luteal phase was in agreement with previous studies [1, 29] and might be related to the presence of edema in the myometrium [30] and higher interstitial fluid content in the endometrium [31] during the luteal phase. The effect was most pronounced in the endometrial layer, which fits, as this is the hormonally most responsive tissue undergoing cyclic remodeling. The observed increase in endometrial thickness in intra-individual analysis from the follicular to luteal phase fits the cyclic variations observed with ultrasound. The significant increase in endometrial D values during the luteal phase might be linked to microvascular changes in the endometrium during the luteal phase, concretely endometrial thickening, blood-vessel-rich arterioles, and enlarged uterine glands [32]. Oral contraceptives and intrauterine devices are commonly used for the management of adenomyosis-related symptoms such as dysmenorrhea [33] and can therefore not be excluded. Previous studies have demonstrated that hormonal contraceptives reduce uterine volume and uterine blood flow [34], potentially explaining the here-observed reduced ADC and D in participants.

The low inter-annotator and intra-annotator agreement observed for the endometrial F and D* parameters could be attributed to variability in delineating a relatively small anatomical region, which can increase the instability of perfusion-related parameters. Motion correction did not substantially affect IVIM parameter estimates in the myometrium and endometrium. However, the observed reduction in fitting stability in the junctional zone, likely due to its thinner anatomical structure, calls for more advanced motion correction techniques.

The capacity to quantify layer-specific diffusion metrics without bowel preparation at 0.55 T addresses a significant gap in adenomyosis assessment and provides quantifiable biomarkers that may clarify diagnosis in morphologically equivocal cases. The significantly lower diffusivity in patients receiving hormonal therapy suggests potential for longitudinal monitoring of treatment response independent of symptom resolution. Anterior–posterior myometrial variation aligns with known heterogeneity in adenomyosis presentation and may provide insight into patterns of disease distribution for future clinical studies. These findings are preliminary and require further validation in independent cohorts before clinical implementation.

The large cohort size of over 100 women is a strength of this study, along with the inclusion of two different models to offer a range of quantitative parameters. This study has, however, some limitations. First, the self-reported menstrual cycle time point of the women was employed, potentially leading to imprecise phase definition. Second, the cohort of participants with adenomyosis was diverse in the exact phenotype of disease, ranging from focal to diffuse adenomyosis, introducing heterogeneity in the quantitative results and contributing to a potentially more complex interpretation of the diffusivity results. The small size of this cohort contributes to this limitation. Expert radiologist classification was performed to counter this limitation to enable sub-cohort analysis. The different paths to the original diagnosis however remain a limitation. Limited intra-individual analysis of healthy participants was performed. Matched recruitment across different menstrual phases for both healthy and adenomyosis participants would have been valuable to better characterize dynamic changes. However, within the adenomyosis group, the majority of participants were receiving hormonal therapy due to symptom-related clinical management, limiting the feasibility of phase-matched comparisons. The use of the 0.55-T scanner, while carrying essential advantages in terms of reduced distortions for the EPI-based diffusion sequence, reduces SNR. While partly addressed with the performed denoising, it remains a limitation to the accuracy of the quantitative results.

Future studies aim to include balanced recruitment across menstrual phases, potentially employing physician-based classification of the exact menstrual phase, as well as increased recruitment of currently under-represented subtypes of adenomyosis. As a further step, the effect of the influence of uterine position and body mass index will be included. Expansion to further uterine pathologies such as malignant conditions enable further characterization of microstructural alterations and their physiological relevance.

Quantitative diffusion and IVIM imaging at 0.55 T without bowel preparation and antispasmodics demonstrate potential for distinguishing adenomyosis from healthy uteri. These functional biomarkers hold potential for understanding gynecologic disease pathophysiology and for monitoring responses to clinical and hormonal therapies.

## Supplementary information

**Additional File 1: Fig S1:** Violin plots of diffusion parameters apparent diffusion coefficient (ADC), diffusivity (D) and pseudo-diffusivity (D*) and perfusion fraction (f) across the three cohorts for all three uterine layers. The central white line indicates the mean value, while the width of the plot reflects the distribution density of participants within that value range. **Fig S2:** Anterior-Posterior analysis of the myometrium and junctional zone for three participants groups showing the apparent diffusion coefficient (ADC), diffusivity (D) and perfusion fraction (f). *p < 0.05 compared with healthy controls and ^†^*p* < 0.05 compared with participants with adenomyosis. **Fig S3:** Plots showing ADC and IVIM parameter variations in adenomyosis cases categorized according to MRI-based adenomyosis classification. The “Other” category includes cases that could not be assigned to a specific MRI subtype but demonstrated imaging features consistent with adenomyosis. **Fig S4:** Bland–Altman plots to evaluate the robustness of diffusion quantification for each uterine layer and diffusion parameter across cohorts between the repeated scans. The bold line represents the mean difference, and the dotted lines indicate the 95% limits of agreement (±1.96 standard deviations). **Fig S5:** IVIM model fitting and stability before and after motion correction registration, showing fitted curves in comparison with the measured averaged diffusion signal across the cohort. Derived parameters, diffusivity (D), pseudo-diffusivity (D*), perfusion fraction (f) and corresponding R² values are provided for each case. **Table S1** Mean Diffusion and IVIM parameter values (Hormonal contraceptive users). **Table S2** Inter-annotator and Intra-annotator variability of ADC and IVIM parameters. **Table S3** Percentage of Variability in ADC and IVIM Metrics.

## Data Availability

The data are available from the authors upon reasonable scientific interest in anonymized form. Processing scripts are equally available.

## Abbreviations

ADC: Apparent diffusion coefficient
D: Diffusivity
D*: Pseudodiffusivity
DWI: Diffusion-weighted imaging
EPI: Echo-planar imaging
f: Perfusion fraction
ICC: Intraclass correlation coefficient
IVIM: Intravoxel incoherent motion
SNR: Signal-to-noise ratio
