# Supplementary material for: Diving deep into uterine layers: ADC and IVIM at 0.55-T MRI
Source: Eur Radiol Exp. 2026 Sep 17;10:134. doi: 10.1186/s41747-026-00815-x (PMC13586134; doi:10.1186/s41747-026-00815-x)
Supplement: Supplementary file 1 [file 41747_2026_815_MOESM1_ESM.pdf]

Diving deep into uterine layers: ADC and IVIM at 0.55-T MRI

ELECTRONIC SUPPLEMENTARY MATERIAL

Supplementary Figure

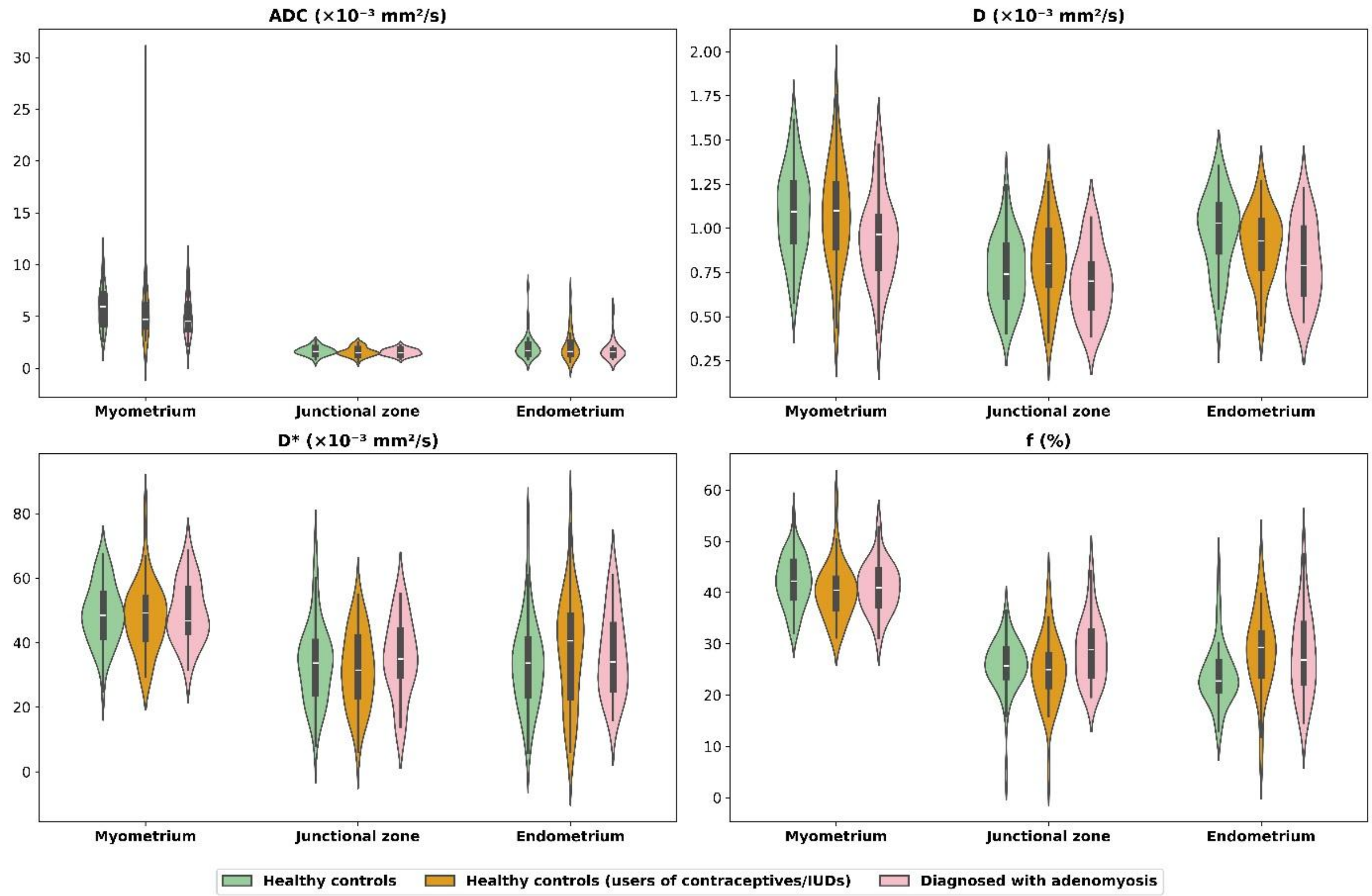

Fig S1: Violin plots of diffusion parameters apparent diffusion coefficient (ADC), diffusivity (D) and pseudo-diffusivity (D*) and perfusion fraction (f) across the three cohorts for all three uterine layers. The central white line indicates the mean value, while the width of the plot reflects the distribution density of participants within that value range.

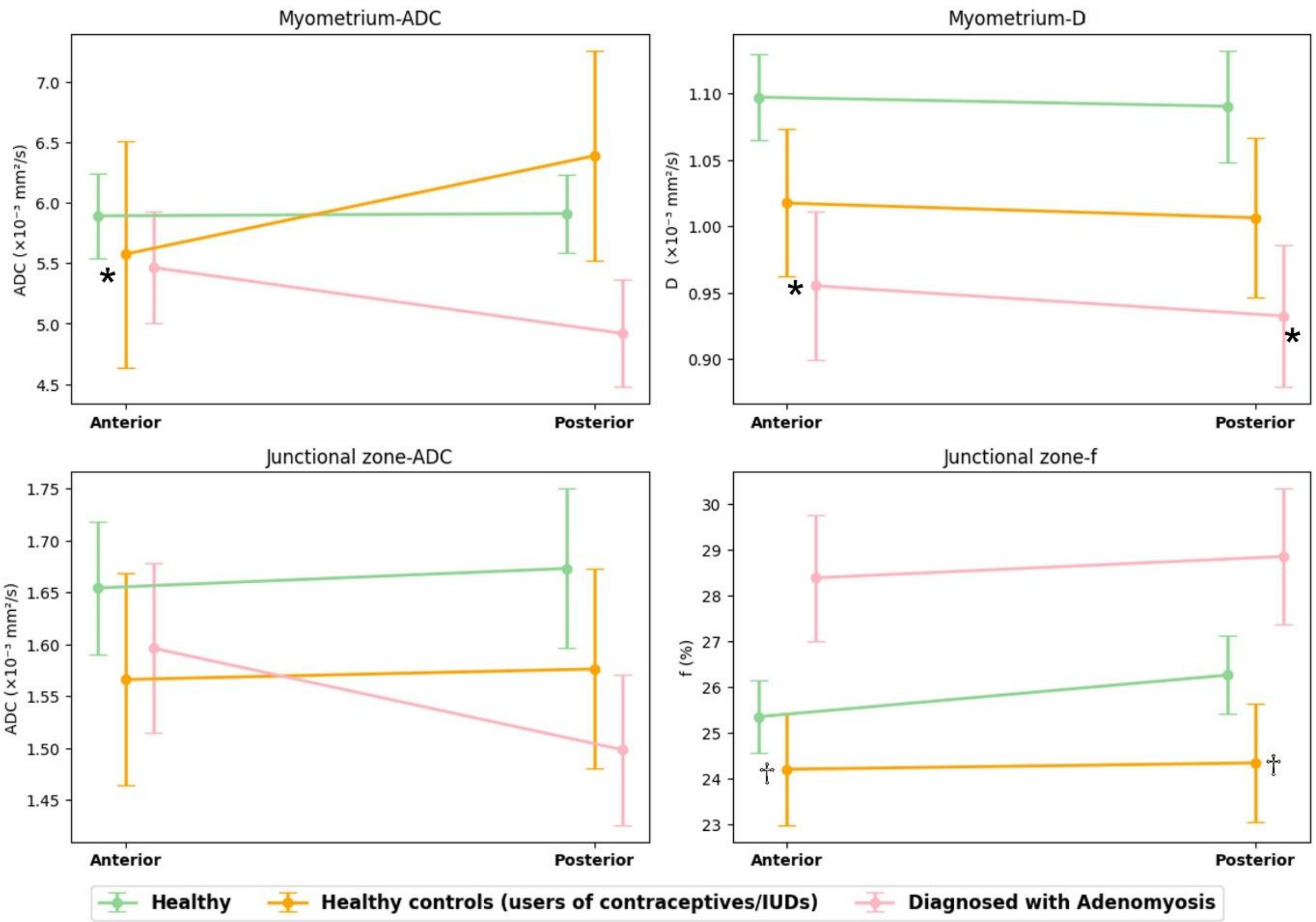

Fig S2: Anterior-Posterior analysis of the myometrium and junctional zone for three participants groups showing the apparent diffusion coefficient (ADC), diffusivity (D) and perfusion fraction (f). *p<.05 compared with healthy controls and †p<.05 compared with participants with adenomyosis.

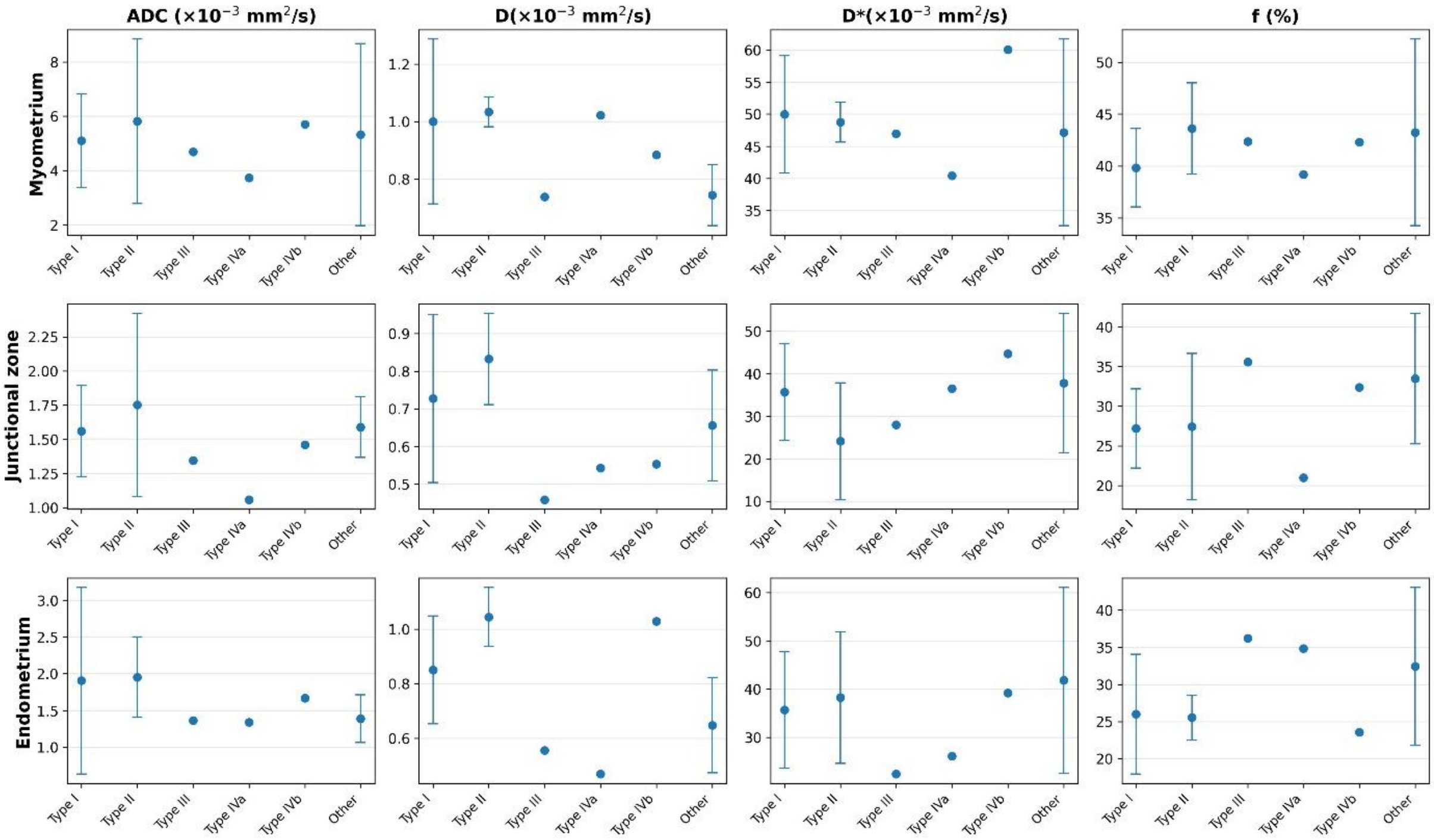

Fig S3: Plots showing ADC and IVIM parameter variations in adenomyosis cases categorized according to MRI-based adenomyosis classification. The “Other” category includes cases that could not be assigned to a specific MRI subtype but demonstrated imaging features consistent with adenomyosis.

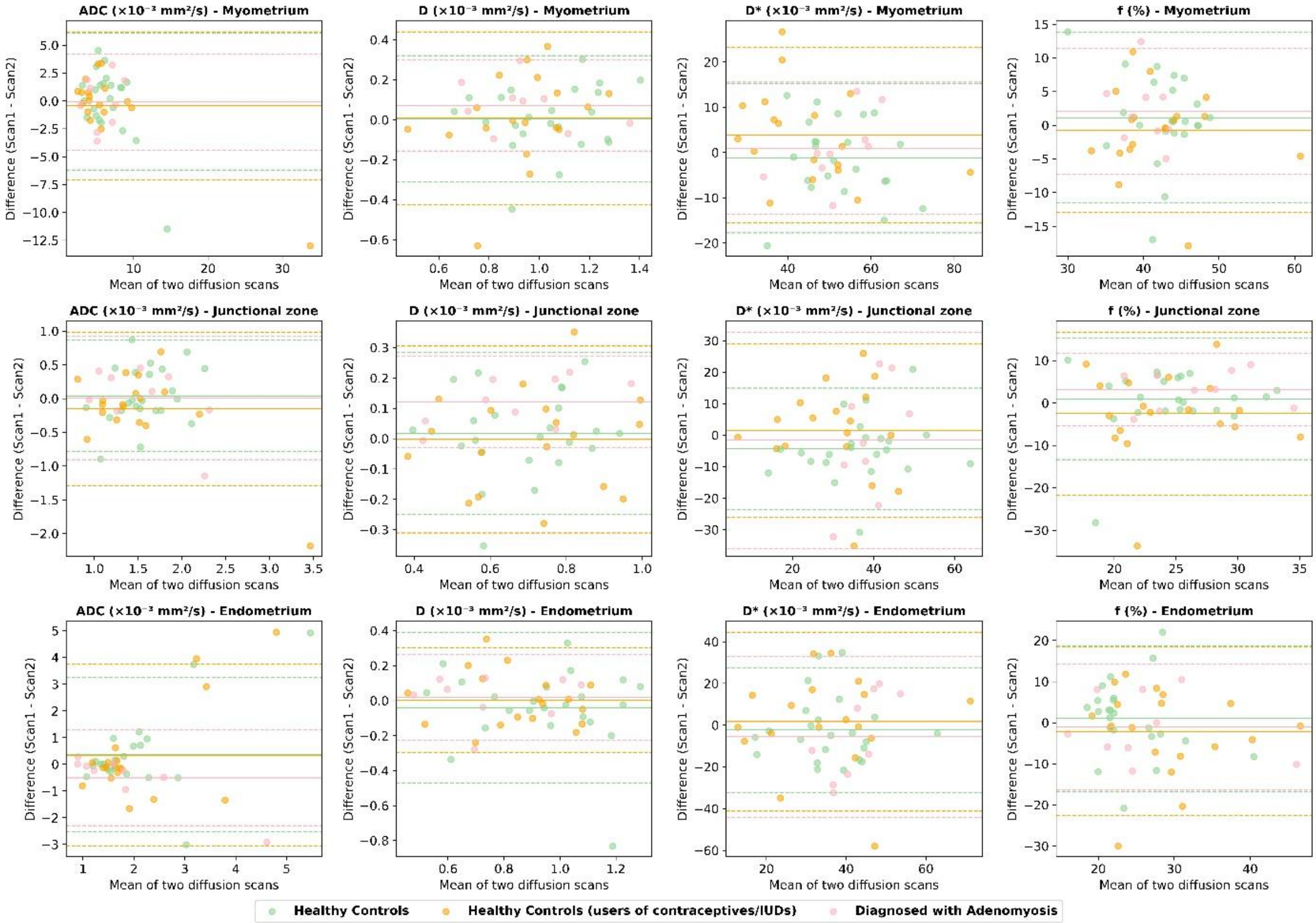

Fig S4: Bland–Altman plots to evaluate the robustness of diffusion quantification for each uterine layer and diffusion parameter across cohorts between the repeated scans. The bold line represents the mean difference, and the dotted lines indicate the 95% limits of agreement (±1.96 standard deviations)

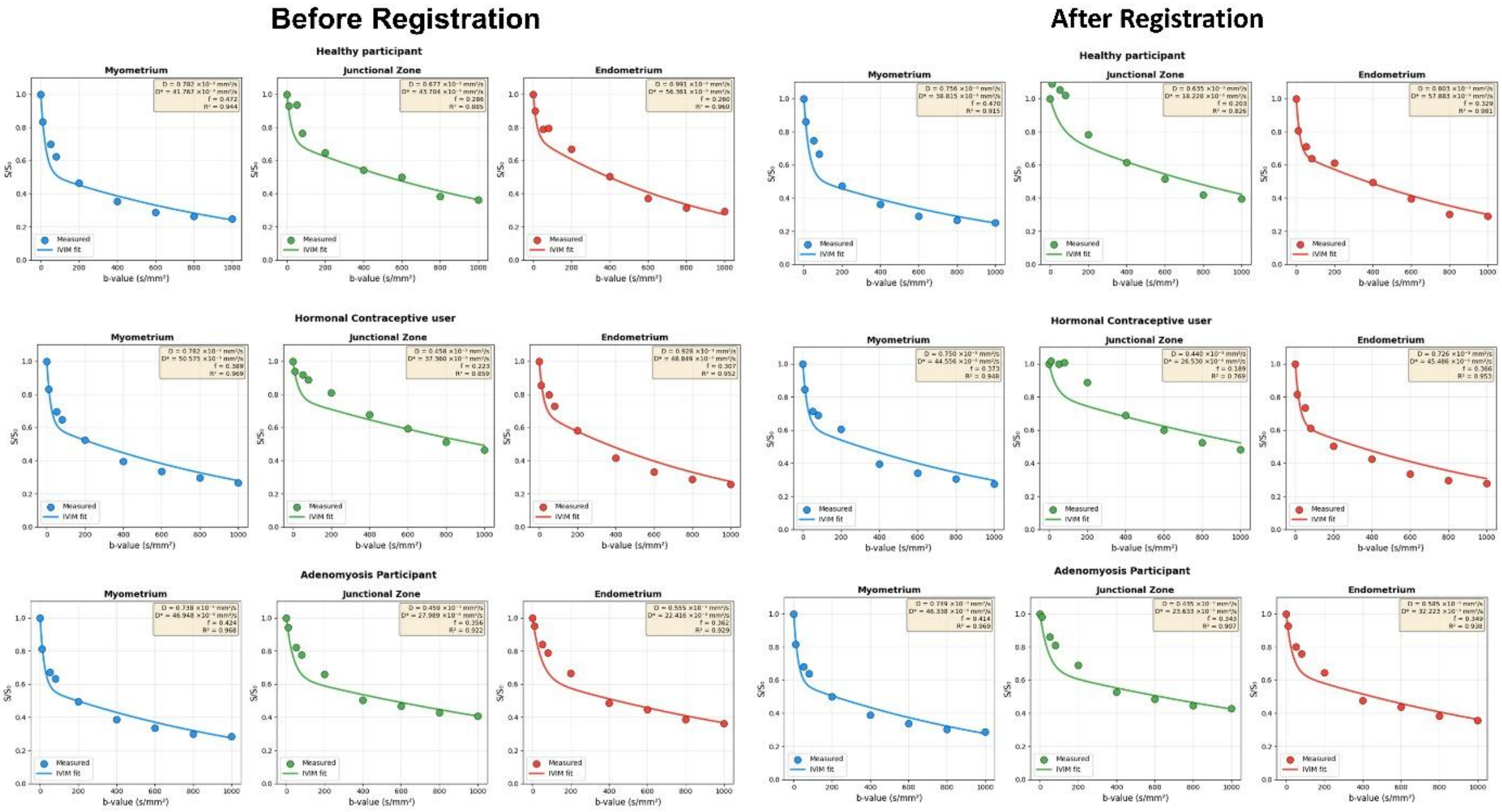

Fig S5: IVIM model fitting and stability before and after motion correction registration, showing fitted curves in comparison with the measured averaged diffusion signal across the cohort. Derived parameters, diffusivity (D), pseudo-diffusivity (D*), perfusion fraction (f) and corresponding R^2 values are provided for each case.

Supplementary Table

Table S1 Mean Diffusion and IVIM parameter values (Hormonal contraceptive users)

Value	ADC	D	D*	f
**Myometrium**				
Regular bleeding	4.8[27.2-2.7]	1.1±0.3	48.4±12.1	39.9±6.1
Amenorrhea	4.4[9.4-2.9]	0.9±0.3	47.2±7.9	40.7±4.4
**Junctional zone**				
Regular bleeding	1.6±0.4	0.9±0.2	30.4±11.6	24.7±7.3
Amenorrhea	1.5±0.4	0.7±0.2	33.1±11.3	24.6±5.4
**Endometrium**				
Regular bleeding	2.0±1.1	0.9±0.2	31.0±15.3	27.8±8.1
Amenorrhea	1.7[7.2-1.1]	0.8±0.2	**45.7±13.1****	29.7±7.0

** signifies p<0.01

Table S1: ADC and IVIM parameter values for hormonal contraceptive users, divided based on regular bleeding and amenorrhea.

Table S2 Inter-annotator and Intra-annotator variability of ADC and IVIM parameters

Parameters	Inter-annotator ICC (95% CI)	Intra-annotator ICC (95% CI)
**Healthy Controls (n=5)**		
ADC Myometrium	0.92 [0.52, 0.99]	0.91[0.51, 0.99]
ADC Junctional Zone	0.80 [0.09, 0.98]	0.77[0.01, 0.97]
ADC Endometrium	0.93 [0.58, 0.99]	0.98[0.87, 1.0]
D Myometrium	0.98 [0.88, 1.0]	0.98[0.92, 1.0]
D Junctional Zone	0.98 [0.9, 1.0]	0.98[0.97, 1.0]
D Endometrium	0.92 [0.55, 0.99]	0.97[0.84, 1.0]
D* Myometrium	0.99 [0.93, 1.0]	0.98[0.92, 1.0]
D* Junctional Zone	0.98 [0.93, 1.0]	0.99[0.96, 1.0]
D* Endometrium	0.93 [0.61, 0.99]	0.95[0.69, 0.99]
F Myometrium	0.96 [0.77, 1.0]	0.97[0.81, 1.0]
F Junctional Zone	0.92 [0.56, 0.99]	0.77[0.02, 0.97]
F Endometrium	0.41 [-0.51, 0.92]	0.93[0.59, 0.99]
**Hormonal Contraceptive users (n=5)**		
ADC Myometrium	0.98 [0.92, 1.0]	0.98[0.88, 1.0]
ADC Junctional Zone	0.71[-0.1,0.97]	0.49[-0.43, 0.93]
ADC Endometrium	0.98 [0.89, 1.0]	0.98[0.88, 1.0]
D Myometrium	0.95 [0.7, 0.99]	0.98[0.88, 1.0]
D Junctional Zone	0.98 [0.92, 1.0]	0.96[0.72, 1.0]
D Endometrium	0.92 [0.53, 0.99]	0.97[0.81. 1.0]
D* Myometrium	0.97[0.8, 1.0]	0.96[0.75, 1.0]
D* Junctional Zone	0.98[0.87, 1.0]	0.95[0.68, 0.99]
D* Endometrium	0.92[0.52, 0.99]	0.95[0.72, 1.0]
F Myometrium	0.97 [0.85, 1.0]	0.98[0.92, 1.0]
F Junctional Zone	0.90 [0.46, 0.99]	0.93[0.62, 0.99]
F Endometrium	0.97 [0.85, 1.0]	0.98[0.88, 1.0]
**Adenomyosis (n=5)**		
ADC Myometrium	0.92 [0.56, 0.99]	0.96[0.79, 1.0]
ADC Junctional Zone	0.88 [0.38, 0.99]	0.80[0.1, 0.98]
ADC Endometrium	0.95 [0.73, 1.0]	0.99[0.96, 1.0]
D Myometrium	0.98 [0.97, 1.0]	0.99[0.96, 1.0]
D Junctional Zone	0.98 [0.94, 1.0]	0.98[0.87, 1.0]
D Endometrium	0.96 [0.78, 1.0]	0.95[0.69, 0.99]
D* Myometrium	0.98 [0.94, 1.0]	0.98[0.94, 1.0]
D* Junctional Zone	0.95 [0.75, 1.0]	0.87[0.31,0.98]
D* Endometrium	0.96 [0.8, 1.0]	0.57[-0.35, 0.94]
F Myometrium	0.94 [0.64, 0.99]	0.81[0.13, 0.98]
F Junctional Zone	0.91 [0.5, 0.99]	0.96[0.72, 1.0]
F Endometrium	0.97 [0.83, 1.0]	0.98[0.87,1.0]

Table S2: Inter-annotator and Intra-annotator agreement of ADC and IVIM parameters in the three uterine layers, shown for each study group. ICC represents intraclass correlation coefficient

Table S3 Percentage of Variability in ADC and IVIM Metrics

Parameters	Percentage of Variability $100 * \frac{1}{N}\sum_{i=1}^{N}\frac{\lvert BeforeRegistration_i - AfterRegistration_i \rvert}{(BeforeRegistration_i + AfterRegistration_i)/2}$
**Healthy Controls (n=5)**	
ADC Myometrium	14.85%
ADC Junctional Zone	28.26%
ADC Endometrium	44.43%
D Myometrium	10.15%
D Junctional Zone	9.46%
D Endometrium	10.16%
D* Myometrium	3.9%
D* Junctional Zone	59.18%
D* Endometrium	28.43%
F Myometrium	2.39%
F Junctional Zone	34.20%
F Endometrium	31.57%
**Hormonal Contraceptive users(n=5)**	
ADC Myometrium	7.93%
ADC Junctional Zone	14.08%
ADC Endometrium	56.89%
D Myometrium	3.18%
D Junctional Zone	5.81%
D Endometrium	16.56%
D* Myometrium	5.79%
D* Junctional Zone	54.47%
D* Endometrium	10.30%
F Myometrium	3.10%
F Junctional Zone	19.10%
F Endometrium	25.90%
**Adenomyosis (n=5)**	
ADC Myometrium	19.27%
ADC Junctional Zone	11.18%
ADC Endometrium	17.24%
D Myometrium	5.34%
D Junctional Zone	4.96%
D Endometrium	12.22%
D* Myometrium	12.45%
D* Junctional Zone	27.34%
D* Endometrium	39.11%
F Myometrium	6.17%
F Junctional Zone	9.34%
F Endometrium	22.51%

Table S3: Percentage of variability in ADC and IVIM metrics in the uterine layers before and after motion correction registration for each study group.
